# Bone Quality in Diabetes

**DOI:** 10.3389/fendo.2013.00072

**Published:** 2013-06-14

**Authors:** Mitsuru Saito, Keishi Marumo

**Affiliations:** ^1^Department of Orthopaedic Surgery, Jikei University School of Medicine, Tokyo, Japan

**Keywords:** collagen cross-links, advanced glycation end products, pentosidine, osteoporosis, diabetes, bone quality

## Abstract

Diabetes is associated with increased risk of fracture, although type 2 diabetes is characterized by normal bone mineral density (BMD). The fracture risk of type 1 diabetes increases beyond an explained by a decrease of BMD. Thus, diabetes may be associated with a reduction of bone strength that is not reflected in the measurement of BMD. Based on the present definition, both bone density and quality, which encompass the structural and material properties of bone, are important factors in the determination of bone strength. Diabetes reduces bone quality rather than BMD. Collagen cross-linking plays an important role in bone strength. Collagen cross-links can be divided into lysyl hydroxylase and lysyl oxidase-mediated enzymatic immature divalent cross-links, mature trivalent cross-links, and glycation- or oxidation-induced non-enzymatic cross-links (Advanced Glycation End-products: AGEs) such as pentosidine. These types of cross-links differ in the mechanism of formation and in function. Not only hyperglycemia, but also oxidative stress induces the reduction in enzymatic beneficial cross-links and the accumulation of disadvantageous AGEs in bone. In this review, we describe the mechanism of low bone quality in diabetes.

Diabetes itself is associated with increased risk of fracture (Vestergaard, 2007), although type 2 diabetes is often characterized by normal or high bone mineral density (BMD). Thus, diabetes may be associated with a reduction of bone strength that is not reflected in the measurement of BMD.

According to the recent definition of bone strength, BMD, and quality in terms of tissue material properties are important determinants. Bone matrix consists of a two-phase composite material in which the mineral phase provides stiffness and collagen fibers provide tensile strength, ductility, and toughness. Bone tissue material properties are regulated by bone tissue turnover rate, cellular activity, and the levels of oxidative stress and glycation (Seeman and Delmas, 2006). Collagen enzymatic and non-enzymatic cross-link formation in bone affect directly not only the mineralization process, but also bone strength. Impaired enzymatic cross-linking and/or an increase in non-enzymatic cross-links, pentosidine (Pen), which is surrogate marker of advanced glycation end products (AGEs), in bone collagen have been proposed as a major cause of bone fragility in aging, osteoporosis, and diabetes mellitus (Robins and Bailey, 1977; Knott and Bailey, 1998; Satio and Marumo, 2010). This review will summarize the mechanism of formation of cross-linking in bone collagen and important aspects of alterations in collagen cross-link formation, in diabetes.

## Cross-Link Formation in Bone Collagen

Type I collagen is the most abundant protein component in bone matrix. Type I collagen consists of two non-helical regions called telopeptides at the amino-terminal (N) and carboxyl-terminal (C) and a central triple helical region. Collagenous plays important roles in tissue mechanical strength and this is attributed to the formation of intermolecular cross-linking formation between the adjacent collagen molecules.

Stabilization of newly formed collagen fibers is initially achieved by the formation of covalent cross-links between neighboring collagen molecules. Collagen cross-links can be divided into two types. One is lysyl oxidase (LOX)-mediated cross-linking (enzymatic cross-links) and glycation or oxidation-induced AGEs cross-linking. These two types vary in the mechanism of formation and display functional differences.

### Enzymatic cross-links of collagen

**Figure 1:**
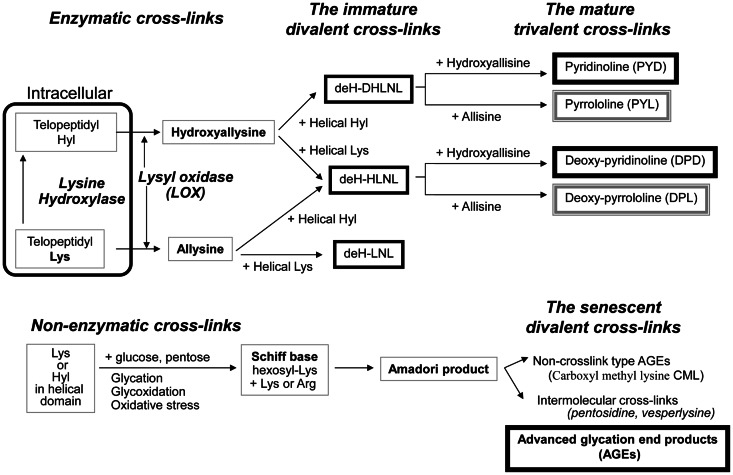
**The pathway of collagen crosslink formation Lysine hydroxylase and lysyl oxidase regulate reaction is the first step or enzymatic cross-linking**. The first step for AGEs formation is the non-enzymatic glycation, oxidation, or glycoxidation between the helical Lys or Hyl residue and sugar. de-H-DHLNL: dehydro-dihydroxylysinonorleucine, de-H-HLNL: dehydro-hydroxylysinonorleucine, de-H-LNL: dehydro-lysinonorleucine, CML: carboxyl methyl lysine.

Enzymatic cross-link formation in bone collagen is regulated by the action of two types of enzymes: the lysine hydroxylases (Procollagen-lysine, 2-oxyglutarate, 5-dioxigenase, PLOD, LH) and LOX. Lysine hydroxylases regulate tissue specific enzymatic cross-link patterns intracellularly (Robins and Bailey, 1977; Bank et al., 1999; Uzawa et al., 1999; Saito et al., 2004a). After intracellular determination of the degree of hydroxylation of lysine (Lys), collagen molecules are secreted into the extracellular space. LOX only acts on extracellular collagen fibers and binds to specific Lys or hydroxylysine (Hyl) residues in the telopeptides to control total amount of enzymatic cross-linking (Figure 1).

The first step of cross-link formation extracellularly is conversion of ε-amino groups of specific for cross-linking site such as Lys and Hyl residues located in the telopeptide domains into the molecule aldehyde to give allysine and hydroxyallysine, respectively (Figure 1). This reaction occurs via the action of LOX following extracellular aggregation of collagen molecules into fibers (Eyre et al., 1984). An excessive formation of enzymatic cross-links does not occur in the physiological mineralization process *in vivo* (Eyre et al., 1988; Yamauchi and Katz, 1993; Saito et al., 1997) and *in vitro* (Kuboki et al., 1992; Uzawa et al., 1999; Saito et al., 2003, 2004a) because the total amount of enzymatic cross-linking in bone is strictly regulated by the expression of LOX. Four LOX like-proteins (LOXL1-4) have been identified although their function and tissue specificity remain unclear (Atsawasuwan et al., 2005). Pyridoxal phosphate (vitamin B6) acts as an essential co-factor of LOX (Bird and Levene, 1982; Wang et al., 1996). We showed that vitamin B6 deficiency in rats resulted in a 25% reduction in LOX mediated cross-link formation in bone compared to regularly fed rats (Fujii et al., 1979). The other positive regulatory factors of LOX are reported. Various growth factors acting in bone formation such as transforming growth factor beta (TGF-β) (Feres-Filho et al., 1995), connective tissue growth factor (CTGF) (Hong et al., 1999), and insulin-like growth factor I (IGF-I) (Reiser et al., 1996). Estrogen is also important regulatory factor of LOX expression. Ovariectomy in mouse led to decrease the activity of LOX down to 25% after OVX, but this reduced activity was completely rescued by estradiol injection (Ozasa et al., 1981). Furthermore, aging-related reduction in estrogen correlates to the activity of LOX in bone (Sanada et al., 1978). In contrast, the negative regulatory factors of LOX are reported. Fibroblast growth factor (FGF) (Feres-Filho et al., 1996), prostaglandin E_2_ (Saito et al., 2004b), and tumor necrosis factor-alpha (TNF-alpha) (Rodríguez et al., 2008) suppresses LOX gene expression and enzymatic activity. In terms of osteoblastic activity, deficient enzymatic crosslink formation by beta-aminopropionitrile (BAPN) treatment hampers osteoblastic differentiation (Turecek et al., 2008). These results indicate that proper enzymatic cross-link formation is essential for osteoblastic differentiation. Homocysteine (Hcys) thought to interfere with enzymatic cross-link formation via a reduction in gene expression and enzymatic activity of LOX (Liu et al., 1997; Raposo et al., 2004). Hcys binds competitively to the aldehydic groups of precursor of cross-linking sites and results in a marked decrease in cross-linking (Kang and Trelstad, 1973). Recently, mildly elevated plasma homocysteine in the general population was reported to be a independent fracture risk (Yang et al., 2012). This plausible mechanism is thought to reduce bone quality in terms of abnormal collagen cross-link formation (Saito et al., 2006a; Blouin et al., 2009). Hyperhomocysteinemia is a risk factor of arteriosclerosis as well as fracture risk in general population (McLean and Hannan, 2007). Type 2 diabetes also increases plasma level of homocysteine, resulting in arteriosclerosis (Ebesunun and Obajobi, 2012). Thus, hyperhomocysteinemia in diabetes may show an additive adverse effect of bone quality. The precursor of enzymatic cross-linking amino-acids such as the telopeptide Lys aldehydes (allysine) and Hyl aldehydes (hydroxyallysine) in the N-terminal telopeptide and in the C-terminal telopeptide then react and condensate with Lys or Hyl residues in the triple helical region of an adjacent collagen molecule to form divalent immature cross-links called deH-dihydroxylysinonorleucine (DHLNL), deH-hydroxylysinonorleucine (HLNL), and deH-lysinonorleucine (LNL). The divalent cross-links undergo a spontaneous reaction with another telopeptide Lys or Hyl aldehyde to form trivalent mature pyridinium or pyrrole cross-links (Figure 1). The presence of mature cross-links was first suspected when the amount of immature divalent cross-links markedly decreased with advancing age in human bone (Fujii et al., 1976), Mature cross-links are pyridinium cross-links such as pyridinoline (PYD) and deoxypyridinoline (DPD) are formed via the hydroxyallysine pathway. Pyrrole cross-links, such as (pyrrolidine) PYL and deoxy-pyrrolidine (DPL) are also characterized as mature trivalent cross-links (Brady and Robins, 2001; Banse et al., 2002). The proposed mechanism of pyrrole cross-link formation is by condensation of de-HLNL/de-DHLNL reacting with another allysine (Kuypers et al., 1992), or by interaction of two divalent immature cross-links including deH-HLNL (Hanson and Eyre, 1996).

## Advanced Glycation End Products (AGEs, Non-Enzymatic Cross-Links)

Although enzymatic cross-links have the beneficial effects on bone strength, non-enzymatic AGEs cross-linking within collagen fibers deteriorates the biological and mechanical functions of bone (Vashishth, 2007). Pen is well-established as an intermolecular cross-linking type of AGEs in bone (Saito et al., 1997, 2006b, 2008, 2010a, 2011a; Wang et al., 2002; Viguet-Carrin et al., 2008) (Figure 1). While the content of Pen in bone correlates positively to total amount of fluorescent AGEs, the measurement of pen used as a surrogate marker of total AGEs formation (Dong et al., 2011). There is no direct evidence whether other AGEs cross-links, such as vesperlysine (Nakamura et al., 1997) identified from AGEs-induced bovine serum albumin, non-fluorescent component-1 (NFC-1) identified from aorta (Sims et al., 1996), and glucosepane (Sell et al., 2005) are formed in bone collagen. Although Pen is just one of many AGEs in bone, Pen measurement in bone is a common quantification method as Pen can be easily and precisely quantified in small specimens by HPLC (Bank et al., 1997; Saito et al., 1997; Viguet-Carrin et al., 2009). This suggests that the pathway of their formation may be similar and Pen may be a useful marker of whole AGEs in bone.

The first step of AGEs cross-link formation of collagen is that the aldehyde of an open chain form of glucose, ketose, or other metabolic intermediate (glyoxal, methylglyoxal, and 3-deoxyglucosone) reacts with a free ε-amino group of collagen-bound Lys or Hyl to form a glycosyl-Lys via Schiff base formation (Robins and Bailey, 1972). The hexosyl Lys or Hyl is stabilized by spontaneous Amadori rearrangement (Figure 1). The Amadori adduct reaction with amino-acids such as Lys or arginine (Arg) in adjacent collagen molecules to form AGEs cross-links between collagen molecules (Monnier et al., 2008). AGEs are formed spontaneously by non-enzymatic glycation or oxidation. AGEs cross-links such as Pen are likely to be formed between helical Lys and Arg without involvement of the telopeptides. It is well-established that increasing inter-helical cross-links such as Pen may contribute to tissue stiffness and resistance to breakdown of the non-helical telopeptides by pepsin (Brennan, 1989; Satio, 1999).

Non-cross-linking types of AGEs such as carboxymethyllysine (CML) as well as cross-linking type of AGEs deteriorate osteoblastic function (Mercer et al., 2007; Ogawa et al., 2007) via the interaction with the cell surface receptor of AGEs, RAGE (Cortizo et al., 2003; Cui et al., 2011; Li et al., 2012). Similar disadvantageous effect of Pen on osteoblastic function is also reported (Sanguineti et al., 2008). In contrast, the effects of AGEs on bone resorption by osteoclast remain controversial. A non-histone nuclear protein, high-mobility group box 1 (HMGB1) is released from bone marrow macrophages in response to, RANKL stimulation. The extracellular HMGB1, through RAGE, in large, regulates osteoclastic actin cytoskeleton remodeling, differentiation, and function (Zhou et al., 2008). Thus, AGEs may increase osteoclast activity (Miyata et al., 1997). The results from *in vitro* study are consistent with other evidence that an increasing of AGEs formation occurs in bone from patients with post-menopausal osteoporosis (Saito et al., 2006a,b) and chronic renal failure (Mitome et al., 2011) with high turnover bone. An opposite conclusion compared to Miyata’s results in an *in vitro* study using rabbit and human mature osteoclasts was reported (Miyata et al., 1997). In contrast, Valcourt et al. (2007) new showed an opposite effect of AGEs on osteoclast activity than Miyata’s study (Miyata et al., 1997). This result seems to explain the increase in Pen formation in bone from type 1 and 2 diabetic animal models (Saito et al., 2006c; Silva et al., 2009) and the patients with type 2 diabetes (Okazaki et al., 1997) with low bone turnover. AGEs formation is regulated by glycemic control, tissue life span, and oxidative stress (Saito et al., 2006a,c, 2010a). The formation of AGEs inhibits competitively enzymatic cross-link formation because AGEs are formed between Lys residues, which are essential sites of enzymatic cross-linking in collagen molecules.

Advanced glycation end products cross-links themselves reduce bone strength without cellular dysfunction. An excessive formation of AGEs cross-links in bone makes collagen fibers brittle, thereby leading to the accumulation of microdamage, which results in deterioration of post-yield properties and toughness (Saito et al., 2008). Induction of AGEs in bovine (Vashishth et al., 2001; Garnero et al., 2006) and human (Tang et al., 2007) bone collagen by ribosylation *in vitro* resulted in a decrease in post-yield strain and energy. Induction of AGEs cross-links in bovine bone collagen by ribosylation for 38 days *in vitro* decreased post-yield strain and energy (Vashishth et al., 2001). A similar result using human cancelous bone incubated with ribose for 7 days (Tang et al., 2007). Garnero et al. (2006) also demonstrated that incubation of fetal bone specimens for 60 days at 37 °C induced a more than 50-fold increase of Pen. In this study, an independent contribution of Pen to bone strength was determined by multivariate regression analyses. Interestingly, an increase in Pen in an incubation time-dependent manner in fetal bone correlated positively to post-yield energy absorption. In this case, Pen accumulation showed a positive effect on bone strength. To explain the reason for these contradictory results, we must consider differences in the level of Pen in bone between fetal bovine bone and elderly human bone. After incubation, the content of Pen in fetal bovine bone did not reach the levels of bone from the elderly people, but more closely matched levels found in young adults (Saito et al., 1997). Thus, the relationships observed in Garnero’s study (Garnero et al., 2006) might represent a correlation between fetal and young bones rather than between adult and elderly. Recently, the content of Pen correlates positively and significantly to total AGEs in bone (Karim et al., 2013). Although Pen is a minor type of AGEs in bone collagen, the estimation of Pen in bone is good surrogate marker of whole AGEs in bone. Because these *in vitro* incubation studies exclude the effect of AGEs on cellular activities and maintain constant bone architecture and BMD, AGEs cross-linking itself may deteriorate bone mechanical strength at a material level. Such disadvantageous effects on bone mechanical properties induced by the excessive accumulation of AGEs cross-links in bone have been shown to correlate with deformation and damage of bone (Wang and Qian, 2006) and microdamage accumulation (Saito et al., 2008). Unfortunately, these studies that investigate the correlation between bone strength and collagen cross-links are limited to acquiring information of AGEs cross-links without accounting for the presence of immature divalent and mature trivalent enzymatic cross-links. Some studies perform only evaluation of AGEs. Thus, we analyzed the correlation between beneficial enzymatic cross-links, disadvantageous AGEs cross-linking Pen, and bone strength by using low bone quality diabetic animal model (Table 1) (Saito et al., 2006c). There was significant association between the enzymatic cross-links and mechanical properties of bone in the WBN/Kob rats. The extent of total enzymatic cross-linking associated modest with stiffness and elastic modulus, but no association with energy absorption and maximum load. In contrast, the content of Pen and the ratio of Pen to total enzymatic cross-links had significant association with not only stiffness and elastic modulus but also energy absorption and maximum load. Interestingly, the ratio of Pen to total enzymatic cross-links was more consistent with bone mechanical properties than with total enzymatic cross-links or Pen. These results indicates that the simultaneously estimation of the immature divalent and mature trivalent enzymatic and non-enzymatic AGEs cross-link is important to understand the role of collagen crosslink formation on bone strength.

**Table 1:** **Association between collagen cross-link parameters and bone mechanical properties in the prediabetic and diabetic WBN/Kob rats**.

	Energy absorption	Stiffness	Elastic modulus	Maximum load
Total enzymatic cross-links	0.229	0.557^*c*^	0.521^*c*^	0.285
Pentosidine	0.312^*a*^	0.677^*c*^	0.579^*c*^	0.448^*b*^
Pentosidine/total enzymatic cross-links	0.332^*a*^	0.699^*c*^	0.603^*c*^	0.460^*b*^

*The values show the correlation coefficients between bone mechanical properties and cross-link parameters such as total enzymatic cross-links and Pentosidine (expressed in mol or mmol/mol of collagen) in the prediabetic and diabetic WBN/Kob rats (6–18 months of age)*.

*The value of total enzymatic cross-links is sum of immature (DHLNL, HLNL, LNL) and mature (Pyr, Dpyr) cross-links*.

*The value of Pentosidine/total enzymatic cross-links is the ratio of the content of Pentosidine to the content of total enzymatic cross-links*.

*Asterisks (a, b, c) indicate p values at p < 0.05, p < 0.01, and p < 0.0001, respectively.Saito et al. (2006c) with permission*.

## Enzymatic and AGEs Cross-Links in Diabetes Mellitus

Although type 2 diabetes is characterized by normal or high BMD, diabetes is associated with an increased risk of fracture (Vestergaard, 2007; Yamamoto et al., 2008, 2009a,b; Schwartz et al., 2011; Leslie et al., 2012). Vestergaard reported (Vestergaard, 2007) that the fracture risk in type 1 diabetes increased significantly much more pronounced than what could be explained from a reduction in BMD. Thus, both type 1 and 2 diabetes may be associated with a reduction of bone strength that does not necessarily reflect BMD. Therefore, it is thought that diabetes deteriorates “bone quality” rather than bone mass and BMD, which suggests impaired material properties in terms of collagen cross-link formation. However, little is known for collagen enzymatic and AGEs cross-links abnormalities in diabetes. Because the metabolism of bone collagen in diabetic patients is influenced by many factors including age of onset, disease duration, insulin status, and glycemic control, we should analyze a suitable animal model for type 1 and type 2 diabetes that is closest to the human disease in terms of bone properties (Okazaki et al., 1997; Silva et al., 2009).

**Figure 2:**
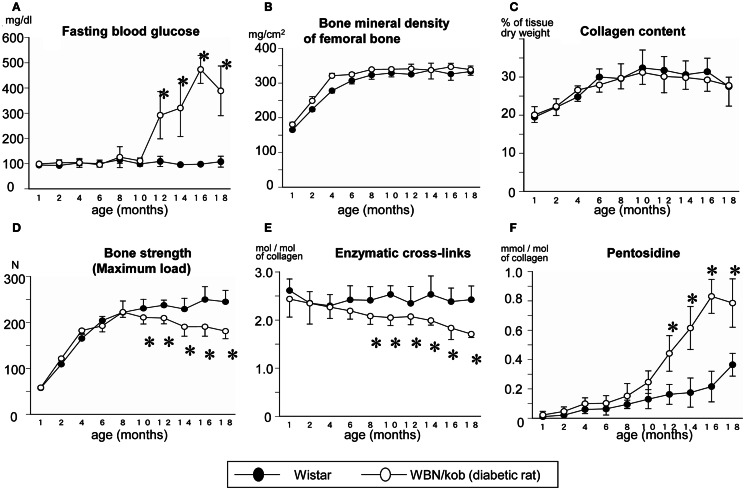
**Serum glucose level (A) and bone mineral density (B), collagen content in bone (C), maximum load (D), total enzymatic cross-link content (the sum of immature divalent and mature trivalent pyridinium crosslinks) (E), and AGEs cross-link Pen content (F) in the non-diabetic Wistar rats (closed circle) and the diabetic WBN/Kob rats (open circle)**. *p < 0.05, vs. the age-matched Wistar rats. (Saito et al. (2006c) with permission).

In terms of type 2 diabetes, we demonstrated that impaired enzymatic cross-links and an excessive formation of AGEs cross-linking Pen in bone collagen decreased bone strength without alteration of collagen content and BMD in spontaneously WBN/Kob rats (Figure 2) (Saito et al., 2006c). In this study, a significant reduction in enzymatic cross-linking even in the subclinical diabetes stage that resulted in a significant decrease in bone strength without changing BMD and AGEs cross-linking (Figure 2). The cause of an impaired enzymatic cross-links in the pre-clinical diabetic stage may be due to a vitamin B6 deficiency, which is essential for the action of LOX (Bird and Levene, 1982). Serum levels of vitamin B6 such as pyridoxal (PL) and pyridoxamine (PM) in the WBN/Kob rats decreased after 6 months of age with age and the serum levels of PL and PM in the WBN/Kob rats were significantly lower than those in the age-matched Wistar rats after 6 months of age. The content of total enzymatic cross-linking associated strongly with serum PL level, whereas the content of Pen had modest association with not only blood glucose but also serum PL and PM levels. It is well known that vitamin B6 are consumed when upregulation of gluconeogenesis takes place in both subclinical and clinical diabetic stages, a latent deficiency of vitamin B6 may provoke impaired LOX-controlled enzymatic cross-linking. We previously reported vitamin B6 deficiency in normal rats reduced enzymatic cross-link formation in bone compared to regularly fed rats (Fujii et al., 1979). An excess of enzymatic cross-link formation cannot occur *in vivo* (Eyre et al., 1988; Saito et al., 1997) and *in vitro* (Kuboki et al., 1992; Uzawa et al., 1999; Saito et al., 2004a) because enzymatic cross-link formation is strictly regulated by LOX activity. Therefore, enzymatic cross-links in physiological conditions may be proportional to the mechanical properties of collagen fibers within a beneficial range without brittleness (Saito et al., 2006c, 2010b, 2011b,c). Therefore, reduction of LOX activity by lathyrogens such as BAPN, pyridoxal phosphate (vitamin B6) deficiency, or copper deficiency induces not only impaired immature divalent cross-links, but also their mature trivalent forms such as pyridinium and pyrrole cross-links, resulting in a decrease of bone strength (Opsahl et al., 1982; Oxlund et al., 1995). Thus, even in a pre-clinical diabetic stage, bone material property is not necessary has sufficient strength.

After the onset of diabetes, there was a steady decrease in enzymatic cross-links and a significant increase in AGEs cross-link, Pen, which was consistent with a significant reduction of bone strength compared to non-diabetic control rats in spite of a lack of change in BMD (Figure 2). As described above, AGEs are thought to be formed between sugar and Lys residues, essential sites of enzymatic cross-linking in collagen, resulting in competitive inhibition of cross-link formation (Monnier et al., 2008). Thus, not only vitamin B6 deficiency, but also accumulation of AGEs may deteriorate enzymatic crosslink formation after the onset of diabetes. A trend toward increased loss in bone quality in terms of impaired enzymatic cross-link formation and/or an excessive accumulation of AGEs cross-links in type 1 and 2 diabetes may lead to accelerated increase of bone fragility, which is independent of BMD.

In terms of type 1 diabetes, Tomasek et al. (1994) reported that streptozotocin-induced type 1 diabetes exhibited an increase in collagen-linked fluorescence (an indicator of AGEs) that was coincident with reduced bone strength, BMD, and bone turnover as estimated by serum levels of osteocalcin compared to control rats. Silva et al. (2009) reported detailed information regarding collagen cross-links, architecture of bone, and bone strength using streptozotocin-induced type 1 diabetic rats. They showed that type 1 diabetes caused trabecular bone loss, a reduction in diaphyseal growth and a marked increase in AGEs cross-link, Pen, without alteration in the content of enzymatic mature pyridinium cross-links or collagen concentration in bone. An increase in AGEs cross-link, Pen, modestly reduced material properties of bone from type 1 diabetic rats.

The major non-collagenous bone matrix protein, osteocalcin, is also important determinant of bone material properties (Tanaka et al., 2011; Poundarik et al., 2012). The molecular deformation and fracture mechanics models, illustrating the important role of osteocalcin in dilatational band formation, and predict that the nanometer scale of tissue organization, associated with dilatational bands, affects fracture at higher scales, and determines fracture toughness of bone (Poundarik et al., 2012).

The osteoporotic hip fracture cases without diabetes shows the significant lower level of osteocalcin in bone compared to non-fractured subjects despite hip osteoarthritis (Tanaka et al., 2011). Interestingly, it is well known that the serum levels osteocalcin in patient with diabetes is significantly lower than the subjects without diabetes (Starup-Linde, 2013). Recently, the inverse correlation between serum levels of sclerostin and osoteocalcin in diabetes was reported despite animal model (Kim et al., 2013; Starup-Linde, 2013). Sclerostin is a Wnt-pathway antagonist produced in the osteocytes (Gennari et al., 2012). The Wnt-pathway promotes osteoblastogenesis and decreases osteoclastogenesis (Manolagas and Almeida, 2007). Since sclerostin is secreted by osteocytes, osteocyte may be a candidate for central role to bone homeostasis in diabetes. Furthermore, AGEs also reduces osteocalcin gene expression in mouse stromal ST2 cells (Okazaki et al., 2012). Thus, increased level of sclerostin as well as hyperglycemia, increased oxidative stress, and AGEs in diabetes may decrease osteocalcin production and accumulation.

To date, there is no suitable biomarker for estimation of bone material properties. Because plasma and urine Pen levels are significantly higher in diabetic patients compared with age-matched healthy subjects (Takahashi et al., 1993; Sugiyama et al., 1998), plasma or urinary levels of Pen may correlate with an independent fracture risk in diabetic patients with low bone quality. Yamamoto et al. (2008, 2009a) reported that a high level of serum Pen or low level of the endogenous secretory receptor for AGEs (esRAGE) acting as decoy receptor of AGEs was independent of prevalent and incident fracture risk in elderly diabetic women. Schwartz et al. (Yamauchi and Katz, 1993) reported that urinary Pen levels were associated with incident clinical fracture or prevalent vertebral fracture, as well as bone turnover markers, in older adults with diabetes in the Health Aging and Body Composition (Health ABC) study. Thus, simultaneous estimation of BMD, bone turnover markers, and a surrogate marker for AGEs, Pen, may be suitable for estimation of fracture risk of diabetic patients.

## Conclusion

Diabetes showing hyperglycemia, oxidative stress, and hyperhomocysteinemia deteriorates bone material properties in terms of collagen post-translational modification such as enzymatic immature and mature cross-links and non-enzymatic AGEs formation. Furthermore, the adverse effects of AGEs on bone cells via the interaction to RAGEs on bone metabolism accelerate bone fragility in diabetes. Proper collagen cross-link formation seems to directly affect the physiological mineralization process and microdamage accumulation. Enzymatic and AGEs cross-link formation are regulated by LOX activity, levels of glycation and oxidative stress, and bone turnover. Future studies needed to elucidate what other regulatory factors of cross-link formation contribute to the process. The recent literature regarding the roles of AGEs allows us to hypothesize that AGEs could explain the molecular link between primary osteoporosis and diabetes. If so, the agent for inhibiting AGEs formation in bone such as raloxifene (Saito et al., 2010a), teripartide (Saito et al., 2011a), and vitamin B6 (Satio et al., 2005) may reduce fracture risk. The development of a non-invasive biomarker that reflects the actual amount of cross-links in matrix, possibly a bone-specific marker, is needed to investigate individual differences in collagen cross-links and susceptibility to bone fractures. Plasma and urinary levels of Pen may be candidates for the estimation of low bone quality.

## Conflict of Interest Statement

The authors declare that the research was conducted in the absence of any commercial or financial relationships that could be construed as a potential conflict of interest.
